# Design and Validation of the Scale TDV-VP Teen Dating Violence: Victimisation and Perpetration [Violencia en Parejas Adolescentes: Victimización y Perpetración] for Spanish Speakers

**DOI:** 10.3390/ijerph18020421

**Published:** 2021-01-07

**Authors:** Encarnación Soriano-Ayala, María Sanabria-Vals, Verónica C. Cala

**Affiliations:** Department of Humanities and Education, Universidad de Almería, Cañada de San Urbano, 04120 Almería, Spain; mariasanvals@gmail.com (M.S.-V.); vcc284@ual.es (V.C.C.)

**Keywords:** teen dating violence, exploratory factor analysis, confirmatory factor analysis

## Abstract

Background: This study offers the design and validation of a scale for measuring violence in adolescent couples from the perspective of victimisation and perpetration for young Spanish speakers. Method: Validation study using exploratory and confirmatory factor analysis with online self-selected sampling and the participation of 422 subjects who met the requirements of being between 13 and 21 years old and currently or recently having a partner. Results: A scale of victimisation in adolescent partner relationships was obtained with 25 items and a scale of violence perpetration with 22 items. Both scales presented five factors: psychological violence, verbal violence, control, jealousy, and sexual violence. Significant differences were found between men and women in victimisation and perpetration of sexual violence. Conclusions: The Teen Dating Violence—Victimisation and Perpetration (TDV)—VP complies with the reliability and validity indices, constituting a very useful instrument for the detection and measurement of violence in Spanish-speaking adolescent couples in health-promotion work.

## 1. Introduction

Teen dating violence (TDV) refers to different types of assault from one partner to the other, intentionally and during courtship [1]. It reflects both the victimisation or violence suffered and the perpetration of the violence or violence committed. Violence between adolescent couples is a major public health and social justice problem [2]. Nowadays, the gravity of this phenomenon has a strong impact on Spanish society, as this type of violence manifests with a frequency and incidence of between two and three times greater than adult couples [3], although the violence recorded in young couples is usually less intense than that recorded in adult couples [4]. Courtship relationships may begin around adolescence, where the individual continues building his or her own maturity. Adolescents involved in TDV are likely to experience both victimisation and perpetration [5], suggesting that violence within relationships may be mutual. Experiences of victimisation in relationships can have long-term consequences, measurable in longitudinal studies, such as increased use of toxic substances and alcohol in episodic situations, depressive symptomatology, suicidal ideation by women, and increase in antisocial behaviour, suicidal ideation and marijuana use in men [6].

Evaluation of TDV is complex as it manifests in various forms, whether physical, psychological, or sexual violence [1]. Additionally, not all evaluation instruments consider all of these aspects, or they consider them under different categories, making a comparison between results complex and confusing [7]. Moreover, the difficulty of this phenomenon is aggravated by young people who catalogue violence when it manifests in a physical and visible way but allow the psychological and sexual aspects to go unnoticed. This fact may be related to the current and large use of social media by young people in the courtship process and during the relationship. In this space, new forms of violence known as cyberbullying or e-violence [4] occur, as reflected in the definition of TDV by the Centers for Disease Control and Prevention [8]. This new digital field recognises abuse, humiliation, insults, threats, control, and isolation, among others, which can be exercised in the relational framework of a teenage couple [9]. Furthermore, the types of psychological violence are predictors of future physical violence [10] and, it has been shown that psychological aggressions usually occur in the initial moments of the relationship in young couples [11]. Therefore, research should be directed to the study of this type of earlier, more subtle, more symbolic, and less visible violence in adolescent and young couples.

The main function of the evaluation tools, in addition to detection, is to approximate the most concrete preventive work possible in each situation. Therefore, the measures should study situations of dating violence to act effectively in health promotion. In this way, investing in research in courtship relationships is investing in the relationships of the future, as some are precursors of the other [12], and while TDV occurs at a stage of learning behaviours, its consolidation takes place in adult life. For this reason, risk predictions would be allowed to be made as they already are, for example, in the Comprehensive Monitoring System in Cases of Gender Violence (VioGén System) of the Secretary of State for Security of the Ministry of the Interior. There are also a number of factors that act as protectors against TDV, such as close friendships and conditional tolerance for violence [13]; or studies that point to a greater risk prediction in experiences of victimisation in the relationship of couples belonging to sexual minority groups [14]. In terms of predicting the perpetration of dating violence, it is important to study factors such as empathy, social support, parental monitoring and school membership [15].

Two of the most commonly used instruments with the validation for the detection and evaluation of TDV in Spanish-speaking youth [16] are the Patient Violence Questionnaire (CUVINO), which has eight factors: detachment, humiliation, sexual assault, coercion, physical aggression, gender-based violence, emotional punishment and instrumental violence [7], and the Conflict in Adolescent Dating Relationships Inventory (CADRI), which includes four factors: physical violence, verbal violence, relational violence and conflict-resolution style [12]. Both questionnaires measure violence in a general way, although the first emphasizes the violence suffered, while the second also contemplates the perpetration of violence. Although the Conflicts Tactics Scale (CTS) and (CTS-2) have been, over time, the most widely used instruments in measuring relational violence, none of them was designed for the young population [7].

Other questionnaires for international use measure more concrete aspects of violence. These questionnaires include the Acceptance of Dating Violence Scale (ADV) [17], Attitudes about Aggression in Dating Situations Scale and the Justification of Verbal/Coercive Tactics Scale (JTVC), which covers aspects related to the justification of both female and male aggression [18]. Further, the Spanish version of the Conflict Resolution Styles Inventory contemplates aspects of positive approach in conflicts [19]; the Dominating and Jealous Tactics Scale allows for the measure of tactics of domination and jealousy [20]; the scale of Cyberviolence in Adolescent Couples (Cib-VPA) [21] and the English version of the Cyberdating Questionnaire [22] focus on jealousy tactics, intrusive control and conduct through social media. The Spanish version of the Dating Violence Questionnaire (DVQ) [23] is a reduction of CUVINO, the Questionnaire of Psychological Violence in Courtship (PDV-Q) is focused on psychological aspects [24], and the Spanish version of physical dating violence [25] focus on physical aspects. From the University of Seville, the Multidimensional Scale of Dating Violence (EMVN) was designed and validated, and although it had the same purpose as this research, sexual violence was little explored, which is the responsibility of sexology.

Therefore, there are few instruments that allow a measurement of TDV that have been validated in the young population and in Spanish, and that contemplate the perspectives of both perpetration and victimisation. The main objective of this study is to design and validate an instrument for the detection of TDV from the perspective of victimisation and perpetration.

## 2. Method

### 2.1. Sample

The research was based on the application of a questionnaire through the Limesurvey platform due to circumstances associated with the health crisis caused by COVID-19. To ensure the inclusion criteria, those who had never had a romantic relationship were asked to refrain from answering. From the 1112 adolescents who answered the questionnaire, the 446 who declared they had had an active relationship in the previous three months were selected. Likewise, those who did not fully complete the TDV-VP questionnaire and those who were outside the age range of 13–21 years were eliminated, as it is from mid-adolescence that individuals develop a greater self-reflective capacity and a complexity in moral statements.

The final sample comprised 422 adolescents between the ages of 13 and 21, of which 73.7% (*n* = 311) identified as female, 26.06% [*n* = 110] as male and 0.24% (*n* = 1) as gender fluid. The mean age of female adolescents was 18.068 (DT = 1.7906) and the mean age of males 17.769 (DT = 1.66102). Regarding the country of birth, 97.4% (*n* = 303) of the female participants were born in Spain, while 2.6% were born in other countries: Romania, England, Germany, Poland, France, Mexico and Cuba. Out of the men, 97.2% (*n* = 107) were born in Spain and the remaining 2.8% in Romania and England. Regarding the sexual orientation variable, 78.8% of women (*n* = 245) reported being heterosexual, 1.6% (*n* = 5) lesbian, gay or homosexual, 15.1% (*n* = 47) bisexual, 1.6 % (*n* = 5) something else and 2.9% (*n* = 9) selected the option ‘I don’t know’. Of the men, 88% (*n* = 97) chose the heterosexual option, 3.7% (*n* = 4) gay or homosexual, 4.5% (*n* = 5) bisexual and 3.7% checked the ‘I don’t know’ option.

### 2.2. Procedure

#### 2.2.1. Transcultural Translation and Dimensions

For the design of the instrument, a bibliographic review of relevant studies was performed. A total of 340 items were collected covering various dimensions: physical violence, psychological violence, sexual violence, harassment/cyberbullying and justification of violence. These items were commented on and analysed by a working group in TDV. A double translation (from English to Spanish and from Spanish to English) was performed, noting that several items matched semantically. It was conducted with the participation of an expert in sexology with English skills and a bilingual person with no knowledge of sexology. Then, to make a first selection in which some repeated items from different instruments were removed, a focus group composed of six experts in sexology and two experts in research methods was made to evaluate the adjustment of the items, leaving a total of 101 items for victimisation and 101 for perpetration of violence.

#### 2.2.2. Validation by Judges

The 202 items were evaluated by 3 psychology experts in sexology and 12 teachers working at TDV, who determined the content validity index (CVI) of each item. A group of judges [26] was selected to analyse each of the test items in the following way: 1 = ‘unnecessary’; 2 = ‘useful’; 3 = ‘essential’. Items with a low CVI were removed, and only those that exceeded the value of 0.51 were selected due to the criterion of having 14 or more experts, according to Lawshe [26]. Additionally, the formulation and wording of each item were evaluated by scoring the clarity from 1 = ‘nothing’ to 4 = ‘many’, rejecting items that did not exceed a minimum of 80% agreement and taking into account the experts’ suggestions for improvement, leaving a total of 108 items, 54 for victimisation and 54 for perpetration.

#### 2.2.3. Validation by Adolescents

A group of ten students participated in the validation of the scale. They were from the third year of secondary education up to the second year of baccalaureate, from a secondary education institute in suburban southern Spain with a large population of immigrants. A semantic validation was made in which terms and expressions were modified, and some items that were considered to be reflected in previous items were deleted, leaving a total of 92 items that were folded into 46 items of victimisation (‘my partner did it to me’) and 46 items of perpetration of violence (‘I did it to my partner’). Items had five answer choices: 0 = ‘never’; 1 = ‘few times’; 2 = ‘sometimes’; 3 = ‘frequently’; 4 = ‘always’).

### 2.3. Field Study

The field study was then conducted with the original version of 46 victimisation items and 46 perpetration items. The survey was applied online by inviting youth associations through Facebook, via email and with the help of teachers from economics, law and education from their courses on the Blackboard platform.

Items with a move away from normal distribution were reviewed. When the outliers were studied through the Mahalanobis test in AMOS, many subjects that needed to be removed appeared, meaning the sample would have reduced by more than 100 subjects. Therefore, we preferred to do a study of normality through a study of the asymmetry and kurtosis of each item, in addition to the multivariate normality also offered by the AMOS program. We decided to remove some items because of the values of asymmetry and kurtosis [27]: V11: ‘My partner threw some object at me with the intention of harming me’, with a value of 5.7 in asymmetry and 34.3 in kurtosis; V13: ‘My partner has physically assaulted me (slap, punch, kick, pulled hair...)’, with a value of 3.7 in asymmetry and 18.85 of kurtosis, which helped us approximate the distribution of the data to normal. Additionally, the answers to some items were in their entirety ‘Never’, making it impossible to execute the factor analysis, as was the case with the V15 variables: ‘My partner has used a knife or weapon against me’ and V19: ’I lost consciousness from a blow my partner gave me in a fight’. Furthermore, the items of physical violence were not answered affirmatively by any young person, so it could not be included in the final validation. In this way, the final questionnaire (Appendix A) would have the most prevalent dimensions among young people, which are those focused on psychological and emotional aspects.

### 2.4. Statistical Analysis

The measurement of the reliability of the instrument was conducted using Cronbach’s alpha test and by constructing validity through exploratory factor analysis (EFA) through the main components, administered for each of the two scales of violence. This technique was applied once discarded using the KMO test (Kaiser–Meyer–Olkin) and Bartlet’s sphericity test, that the correlations between the items constitute an identity matrix. The rotation method used was Promax.

**Figure 1:**
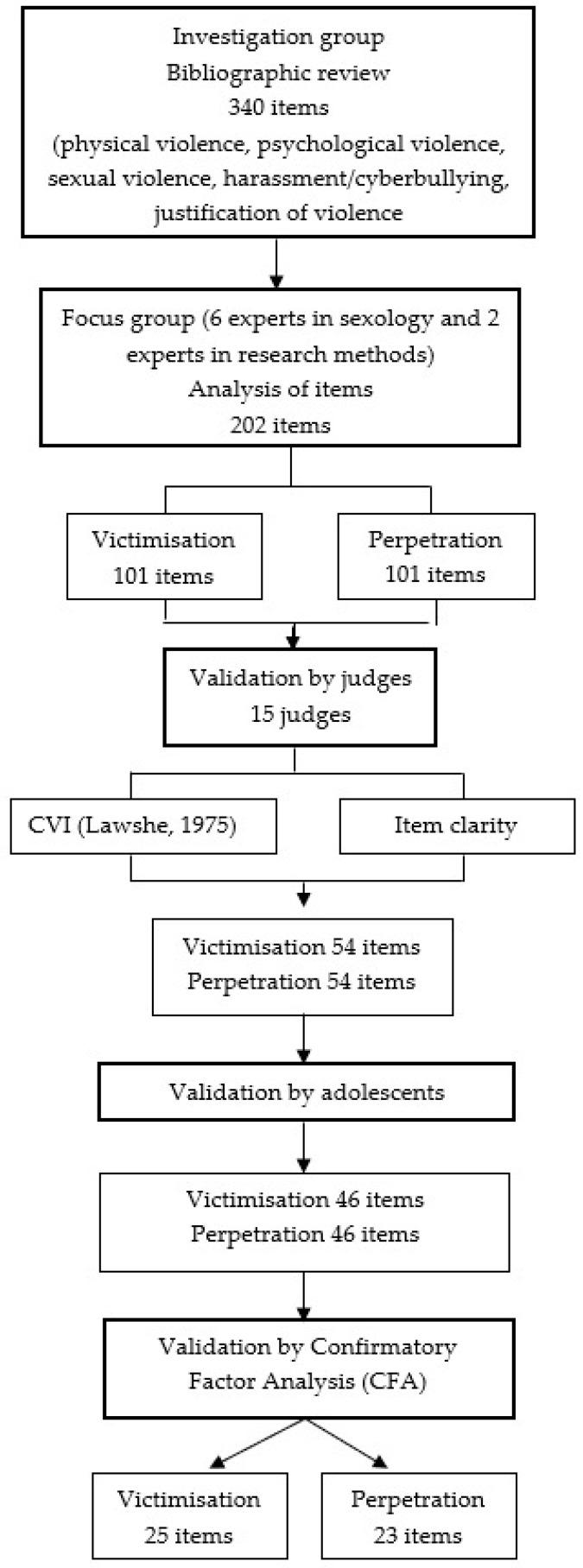
Methodological procedure of the instrument validation.

A confirmatory factorial analysis (CFA) was subsequently conducted via the maximum likelihood method. Although the sample did not present an absolutely normal multivariate distribution, the authors suggested the execution of this analysis [28,29], in addition to other authors who pointed out that this method is robust to non-compliance with this normal multivariate distribution requirement if the variables have a normal univariable distribution [30]. To evaluate the proposed models for victimisation and perpetration, Chi-Cuadrado ratio divided by the degrees of freedom, (CMIN/DF), Incremental Fit Index (IFI), Comparative Fit Index (CFI), Pratio Parsimonious Normed Fit Index (PNFI), Pratio Parsimonious Comparative Fit Index (PCFI), Root Mean Square Error of Approximation (RMSEA), Tucker-Lewis Index (TLI) and NFI were used [31,32]. Figure 1 shows the instrument validation procedure. In addition, the study is completed by calculating the Composite reliability (CR), the average variance extracted, AVE) and the discriminant validity.

Analysis of violence in adolescent relationships was carried out through the Statistical package SPSS 25.0 (IBM, New York, NY, USA) and the AMOS program (IBM, New York, NY, USA).

## 3. Results

For the scale of victimisation, Bartlett’s sphericity test was conducted. It was found that the level of significance (*p* ≤ 0.000) was less than 0.05, with a Chi-square value of 7907.37 (gl = 300) and 0.948 the sample rate of KMO. According to these results, the exploratory factor analysis was performed. Initially, seven factors appeared: one with a single item and another factor with two items. The factorial weights of the items were analysed and studied, regrouped into five factors after eliminating the corresponding ones. Thus, the exploratory factor analysis confirmed the existence of five factors that explain 71.07% of the variance through 25 items. The factors and reliabilities are presented in Table 1. It is suggested that an internal consistency value of 0.6 can be considered acceptable for scales with less than 10 items [33]. For the scale of perpetration of violence, Bartlett’s sphericity test (*p* ≤ 0.000) was conducted, resulting in a Chi-square value of 3570.750 (df-231) and 0.896 the KMO index, so the Exploratory Factor Analysis (EFA) was made with the 22-item scale. Five factors were extracted that explained 57.925% of the variance.

### 3.1. Model Fit: Confirmatory Factor Analysis

The Chi-Cuadrado ratio divided by the degrees of freedom, CMIN/DF, was between values 2 and 5; Incremental Fit Indices (IFIs) and Comparative Fit Indices (CFIs) had a value greater than 0.92; Pratio PNFI and Pratio PCFI had a value greater than 0.7; RMSEA had a value less than 0.07 and TLI and NFI were greater than 0.92 [31].

The results for the victimisation scale obtained a good goodness of fit between the proposed model and the observed data, always complying with the recommendations of the author previously mentioned.

The results for the perpetration scale obtained an acceptable goodness of fit between the proposed model and the observed data. The IFI and CFI incremental adjustment values exceed the value 0.9, and the root of the average approximation quadratic residue (0.066) was even lower than that of the victimisation scale, which is favourable. The rest of the values were good, following the recommendations [31]. This provided two models with five factors whose goodness adjustment rates are presented in Table 2.

**Figure 2:**
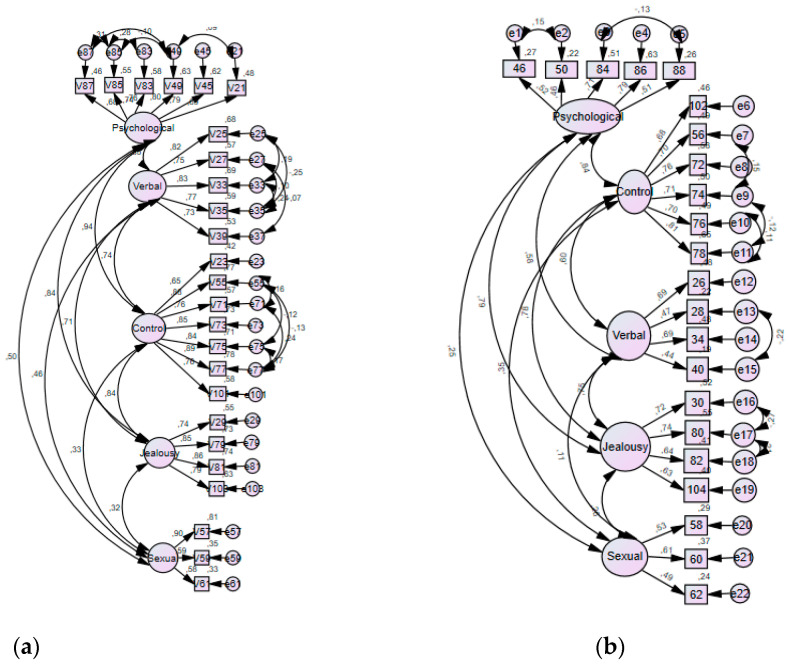
Confirmatory factor analysis path diagram on five proposed dimensions for victimisation (**A**) and perpetration (**B**) scale.

Figure 2 shows the confirmatory factor analysis path diagram on five proposed dimensions for victimisation and perpetration.

Table 3 shows the Standardized Regression Weights (R) and the Square Multiple Correlations (R2) for the two models, victimisation and perpetration. The Square Multiple Correlations (R2) measure the percentage of common variance between the observable variable and its latent variable, and it is advisable that the values are greater than 0.50.

The Square Multiple Correlations in the victimisation scale ranged from the value 0.321 to 0.814. The latent variable “Psychological violence” had two observable variables: “Turning friends against the partner” and “Removing comments, photos or videos of the partner from social networks because they made them jealous”, with lower values but very close to 0.50, for what they were considered adjusted to this latent variable. The observable variables “Insisting on touches that are not pleasant to the partner or that the partner did not want” and “Treating the partner as a sexual object” had a percentage of common variance with the latent variable “Sexual violence” with values of 0.346 and 0.331, respectively. The rest of the observable variables showed a shared common variance with their respective latent variables greater than 0.5.

Regarding the scale of perpetration, the Square Multiple Correlations ranged between 0.191 and 0.678, with seven correlations greater than 0.5. There were three observable variables on the “Psychological violence” scale that shared between 0.2 and 0.3 of the variances with it. In “Verbal violence”, the variable “Feeling that you can’t talk to the partner because she’s almost always mad at me” was the only one that presented the least common variance with its latent variable (0.191). The three observable variables of sexual violence presented low values of common variance with its latent variable. The victimisation scale was the one with the most common variance between the observable variables and their respective latent variables. However, the perpetration scale presented less common variance between the observable variables and the latent variables that compose it.

### 3.2. Composite Reliability, Average Variance Extracted, Convergent and Discriminant Validity

Table 4 shows the average variance extracted (AVE) of each of the victimisation scales studied was greater than 0.5, a critical value [34]. For this reason, it showed a good value in the five latent variables when used as an indicator of convergent validity, so that the constructs explained more than half of the variance of the respective indicators. It did not occur with these factors on the perpetration scale since the only value greater than 0.5 was obtained on the control scale. In the comparison between the square root of AVE (in bold) and the correlation between the scales, only in the sexual variable, both in victimisation and in perpetration, it was higher than the value of the correlation between them, which indicated that the models (victimisation and perpetration) did not show good discriminant validity.

### 3.3. Differences in Sex

By performing the Kolmogorov–Smirnov normality test of the items that made up the scales of victimisation and perpetration (*p* = 0.000), the execution of Mann–Whitney U nonparametric tests for comparisons based on sex justified was performed. As for the comparison by reason of sex, significant differences were found between men and women only in terms of sexual violence. On the one hand, women recognised greater suffering from this violence, while men recognised greater perpetration of violent behaviours within the sexual framework of the relationship of the couple, as demonstrated in Table 5. On the other hand, these differences were considered small due to the value of r de Rosenthal [35] interpreted similarly to Cohen’s d [36], so an effect size between 0.2 and 0.5 would be understood as small; between 0.5 and 0.8 would be moderate, and a value greater than 0.8 would be large [37].

## 4. Discussion

The objective of this study was to design and validate a scale for the detection and measurement of TDV-VP. Due to the results, it is stated that the victimisation scale meets good reliability and validity criteria for application in the Spanish-speaking youth population. In the case of the perpetration scale, the composite reliability had better scores than Cronbach’s Alpha. The advantages of this phenomenon are that the composite reliability is a more complete criterion since it is not influenced by the number of items of the latent variable [34]. However, the perpetration scale obtained worse results than the victimisation scale that may be explained by social desirability [38], so it is important to include this phenomenon in future studies to control its influence. The TDV-VP provides a useful tool for the recognition of victimisation and the perpetration of violence, measuring all five aspects: psychological violence, verbal violence, control, jealousy and sexual violence. The population used in the validation of the instrument did not provide enough information to contemplate a sixth aspect in relation to physical violence, as this is contemplated by instruments such as CADRI (12) or CUVINO [7].

However, the TDV-VP examines sexual violence, which is considered one of the strengths of the scale in considering this type of violence as an indispensable factor in the measurement of TDV. Moreover, this instrument has focused on the violent aspects that are more present in teen dating relationships (psychological, control, jealousy and verbal violence). These types of behaviours are those that manifest themselves in a more masked and subtle way than physical violence and, therefore, they can be more difficult to detect by the adolescent and young population.

Neither of the two scales had good discriminant validity, and it can be explained by the conceptual closeness between the constructs of psychological violence, control, jealousy and verbal violence. These four dimensions presented high correlations with each other, but not with the sexual violence factor. This discriminant validity must be improved in future studies by analysing each dimension in detail.

Sexual violence has been the most controversial dimension according to the results for reasons of differences between the sexes. The results of our study have been directed in the same direction according to reasons of sex [11,39]. Little research has achieved similar results in the character of sexual violence within the TDV [40], with girls showing lower rates of perpetration than boys (3% vs. 10%) and higher rates of victimisation than boys (14% vs. 8%). One factor studied in relation to the prevalence of sexual violence is the consumption of pornography. Exposure to violent pornography was associated with all types of TDV, although some patterns differed by gender [14,41]. Similarly, men who scored higher in what they called ‘male hostility’ and were frequent consumers of pornography were more likely to report sexual coercion [42]. The importance of contemplating sexual violence is indisputable. There is a strong need to address the consequences of shortcomings in the educational system regarding comprehensive sex education, especially from the perspective of equality and sexual diversity.

The absence of significant relationships according to reasons of sex with control, verbal violence, jealousy and psychological violence was considered to be remarkable. Neither men nor women saw a pronounced difference in the experience of victimisation and perpetration of those types of violence. This could be explained by the normalisation and naturalisation of these behaviours within the relationships of adolescents. Verbal–emotional violence [39] is the most likely kind of violence experienced by young people between the ages of 14 and 20. In fact, one of the most frequent types of aggression was insulting or ridiculing in networks, which coincides with the most prevalent behaviour of verbal behaviour [39,43]. The relationship between victimisation and perpetration sheds light on the experience of mutual violence in relationships of couples involved in TDV [5], which can also be explained by the intergenerational transmission theory of violence. It would be interesting to analyse violence factors, such as relationships between adolescents and their parents, from this perspective. Two-way violence could also be explained as self-defence, among other reasons [1]. This bidirectionality of violence would justify the high and moderate correlations of these four dimensions in both victimisation and perpetration. However, in the case of sexual violence, a different phenomenon occurs, and it is related to an imbalance of power between the aggressor and the victim [11] that derives from sexist and patriarchal beliefs and a lack of sexual education.

With the rise in new technologies, new areas where violent acts are committed have emerged. Nowadays, control of one partner over the other has shifted to digital screens. Surveillance and control is one of the most common forms of violence in adolescent relationships, and one of the variables that contribute to the explanation of cyberbullying in dating relationships is jealousy [43], which, under the myth of romantic love, justifies this type of behaviour [39].

Setting the point of view on control across networks depending on sex, it was found that just over a quarter of young people in a current or recent relationship having experienced some form of victimisation of network abuse in the previous year [44]. In addition, women suffer more victimisation than men, especially sexual abuse. One in ten young people acknowledged that they had perpetrated cyber-dating abuse. In contrast, young males were significantly more likely to report cyber-dating sexual abuse. Victims of cyber-dating sexual abuse were seven times more likely to have suffered sexual coercion.

The main limitations of this study lie in the length of the scale for both the validation by judges and for the collection of data of the participants. These may have led to tiredness or exhaustion, which may have influenced the responses. For example, there was a question whose answers were distractors, and it was answered. A possible way to overcome this would be a second validation by judges and a reduction of items. Additionally, the data collection was originally going to be in schools in the province of Almería.

Another limitation of the present study was that the sample was generally female. Because the sample was collected online, the explanation may be given by greater maintenance of privacy by men and greater openness and predisposition to share personal experiences and feelings by women.

The absence of affirmative responses in the items on physical violence, and their disappearance in the validated TDV-VP scale may respond to different explanatory factors. On the one hand, it may be related to a change in the forms of violence experienced in adolescence. It may, in turn, be influenced by the moralisation and social stigmatisation of the issue of intimate partner violence that leads young people to deny the practice of violence (social desirability). On the other hand, it can be greatly affected by the characteristics of the sample participating in the study, which is made up of a group mainly of women, middle class, indigenous, who voluntarily participated in research with a sample through social networks (volunteer bias).

However, due to COVID-19, it was adapted into an online survey, and some 21-year-olds were kept in the sample due to this low participation situation. In this way, despite the fact that the sample of sexual diversity is not wide, it is necessary to study the phenomenon of differences by sex and sexual orientation in future studies. For this reason, a search for a larger homosexual and bisexual sample will be considered in future investigations to address aspects such as intragender violence. With regard to other research, a multicultural sample should be available to analyze the perspectives and beliefs around intimate partner violence and assess the limitations of this questionnaire in a cross-cultural way.

## 5. Conclusions

The study of violence in couple relationships must be adapted to new platforms where violent behaviours occur, such as social media. This investigation has highlighted aspects of violence that occur through physical contact, such as sexual violence, as well as behaviours that are manifested in novel ways, such as control and cyberbullying.

While gender violence is an issue that requires research, education and prevention, the study of domestic violence—which encompasses the experiences of couples outside of relational hegemony—can not be overlooked.

## Figures and Tables

**Table 1:** Reliability of the teen dating violence (TDV)-VP Scale.

Factors	Victimisation Items	α	Perpetration Items	α
Psychological Violence	V21, V45, V49, V83, V85, V87	0.883	V46, V50, V84, V86, V88	0.722
Control	V23, V55, V71, V73, V75, V77, V101	0.927	V56, V72, V74, V76, V78, V102	0.772
Jealousy	V29, V79, V81, V103	0.882	V30, V80, V82, V104	0.772
Verbal Violence	V25, V27, V33, V35, V39	0.874	V26, V28, V34, V40	0.631
Sexual Violence	V57, V59, V61	0.707	V58, V60, V62	0.503

*N* = 422, α de Cronbach.

**Table 2:** Fit indexes for the model of victimisation and perpetration scales tested.

Model	CMIN/DF	IFI	CFI	PNFI	PCFI	TLI	NFI	RMSEA
Victimisation	3.228	0.932	0.932	0.718	0.739	0.914	0.905	0.073
Perpetration	2.809	0.902	0.900	0.696	0.733	0.877	0.855	0.066

CMIN/DF = chi-square fit index divided by degrees of freedom; IFI = Incremental Fit Index; CFI = Comparative Fit Index; PNFI = Parsimonious Normed Fit Index; PCFI = Parsimonious Comparative Fit Index; TLI = Tucker-Lewis Index; NFI = Normed Fit Index; RMSEA = Root Mean Square Error of Approximation

**Table 3:** Factorial weights (R) and Square Multiple Correlations (R^2^) for victimisation and perpetration scales.

		Victimisation		Perpetration	
Factors	Items	R	R^2^	R	R^2^
Psychological Violence	V21: Turning friends against the partner	0.691	0.478	-	-
	V45/V46: Controlling or trying to prevent the partner from doing something they wanted to do with comments	0.786	0.618	0.519	0.270
	V49/V50: Trying not to talk to or see their friends and/or family	0.794	0.631	0.462	0.213
	V83/V84: Not letting the partner chat with some friends and getting angry if they do	0.762	0.580	0.715	0.512
	V85/V86: Removing or blocking friends from the partner’s social media or mobile so that they do not have contact with them	0.752	0.565	0.791	0.626
	V87/V88: Removing comments, photos or videos of the partner from social networks because they made them jealous	0.675	0.456	0.514	0.264
Control V.	V23: Threatening to self-harm	0.648	0.420	-	-
	V55/V56: Checking what the partner does and demanding that he/she tell you where he/she has been	0.862	0.744	0.721	0.520
	V71/V72: Asking the partner where they are every minute of the day	0.762	0.580	0.773	0.597
	V73/V74: Spying on the partner’s communications (phone, emails, social networks...).	0.843	0.710	0.706	0.499
	V75/V76: Checking with friends and family and by other means whether what the partner says about his activities was true	0.851	0.723	0.697	0.485
	V77/V78: Keeping an eye on everything the partner does	0.884	0.782	0.823	0.678
	V101/V102: Trying to gain access to the partner’s social media account	0.763	0.582	0.698	0.488
Verbal V.	V25/V26: Insulting with offensive phrases	0.820	0.673	0.692	0.478
	V27/V28: Insulting or disparaging in front of others	0.754	0.569	0.472	0.222
	V33/V34: Criticising, insulting or shouting	0.826	0.683	0.693	0.481
	V35: Manipulating with lies	0.763	0.583	-	-
	V39/V40: Feeling that you cannot talk to the partner because she’s almost always mad at me	0.730	0.533	0.438	0.191
Jealousy V.	V29/V30: Accusing of flirting with another	0.773	0.598	0.718	0.515
	V79/V80: Getting angry if you see the partner is online and doesn’t answer you right away	0.777	0.604	0.744	0.553
	V81/V82: Being aware of whether the partner is online, on a mobile or connected to social media	0.779	0.608	0.640	0.409
	V103/V104: Getting jealous after reading messages the partner receives on their account or in comments on their photos	0.815	0.664	0.633	0.401
Sexual V.	V57/V58: Forcing sexual activity when the partner did not want to	0.902	0.814	0.535	0.286
	V59/V60: Insisting on touches that are not pleasant to the partner or that the partner did not want	0.588	0.346	0.609	0.371
	V61/V62: Treating the partner as a sexual object	0.575	0.331	0.494	0.244

R = Standardized Regression Weights, R^2^ = Square Multiple Correlations.

**Table 4:** Composite reliability, average variance extracted (AVE), Discriminant validity in victimisation and perpetration of violence.

Factors			Discriminant Validity				
**Victimisation**	**Composite reliability**	**AVE**	**Psychological Violence**	**Verbal Violence**	**Control**	**Jealousy**	**Sexual Violence**
V. Psychological	0.86	0.554	0.750				
V. Verbal	0.82	0.608	0.847	0.780			
Control	0.87	0.648	0.930	0.730	0.801		
Jealousy	0.86	0.620	0.840	0.710	0.877	0.780	
V. Sexual	0.75	0.50	0.460	0.460	0.332	0.322	0.710
**Perpetration**			**Psychological Violence**	**Verbal Violence**	**Control**	**Jealousy**	**Sexual Violence**
V. Psychological	0.81	0.380	0.616				
V. Verbal	0.79	0.350	0.579	0.591			
Control	0.85	0.550	0.832	0.591	0.742		
Jealousy	0.68	0.310	0.791	0.745	0.767	0.693	
V. Sexual	0.74	0.480	0.248	0.113	0.348	0.256	0.556

**Table 5:** Differences in victimisation and perpetration by sex.

**Victimisation**	**Sex**	**N**	**Average Range**	**Z**	**Sig.**	**r**
Sexual Violence	Man Woman Total	110 311 421	190.38 218.29	−2.774	0.006 **	0.13
**Perpetration**	**Sex**	**N**	**Average Range**	**Z**	**Sig.**	**r**
Sexual Violence	Man Woman Total	110 311 421	226.31 205.58	−2.893	0.004 **	0.14

** *p* ≤ 0.01.

## Data Availability

The data presented in this study are available on request from the corresponding author.

## Appendix A. Teen Dating Violence—Victimisation and Perpetration (TDV-VP)

Victimisation

1. Mi pareja no me deja chatear con algunos amigos/as y se enfada si lo hago. (My partner does not let me chat with some friends and he/she gets angry if I do it.)
2. Mi pareja me ha hecho eliminar o bloquear amigos/as de mis redes sociales o de mi móvil para que no tenga contacto con ellos. (My partner has made me delete or block friends from my social networks or from my mobile so that I do not have contact with them.)
3. Mi pareja me ha hecho eliminar comentarios, fotos o vídeos míos en redes sociales porque le ponían celoso/a. (My partner has made me delete comments, photos or videos of me on social networks because they made him/her jealous.)
4. Mi pareja ha tratado de poner a mis amigos en mi contra. (My partner has tried to turn my friends against me.)
5. Mi pareja ha controlado o tratado de impedir con comentarios que yo haga algo que quería hacer. (My partner has controlled or tried to prevent me from doing something I wanted to do with comments.)
6. Mi pareja ha intentado que no hable o vea a mis amigos y/o familiares. (My partner has tried to stop me from talking to or seeing my friends and/or family.)
7. Mi pareja me amenazó con autolesionarse. (My partner threatened to harm herself/himself.)
8. Mi pareja comprueba lo que hago y me exige que le diga dónde he estado. (My partner checks what I do and demands me to tell him/her where I have been.)
9. Mi pareja me pregunta dónde estoy cada minuto del día. (My partner asks me where I am every minute of the day.)
10. Mi pareja ha espiado mis cosas (teléfono, correos, redes sociales…). (My partner has spied on my things (phone, emails, social networks…))
11. Mi pareja ha comprobado por amistades, familiares y otra vía si es cierto que estaba donde le dije que estaba. (My partner has checked with friends, family and another way if it is true that I was where I said that I was.)
12. Mi pareja vigila todo lo que yo hago. (My partner watches everything I do).
13. Mi pareja ha intentado obtener acceso a mi cuenta de red social. (My partner has tried to access my social network account.)
14. Mi pareja me ha acusado de coquetear con otro/a. (My partner has accused me of flirting with someone else).
15. Mi pareja se enfada si ve que estoy en línea y no le contesto enseguida. (My partner gets angry if he/she sees that I am online and I do not answer him/her right away.)
16. Mi pareja está pendiente de si estoy en línea en el móvil o conectado/a en las redes sociales. (My partner is aware of whether I am online on my mobile or connected to social networks.)
17. Mi pareja se pone celoso/a después de leer los mensajes que recibo en mi cuenta o en comentarios en mis fotos. (My partner gets jealous after reading the messages I receive on my account or in comments on my photos.)
18. Mi pareja me ha insultado con frases ofensivas. (My partner has insulted me with offensive phrases.)
19. Mi pareja me ha insultado o menospreciado delante de los demás. (My partner has insulted or belittled me in front of others.)
20. Mi pareja me ha criticado, insultado o gritado. (My partner has criticized, insulted or yelled at me.)
21. Mi pareja me ha manipulado con mentiras. (My partner has manipulated me with lies.)
22. Siento que no puedo hablar con mi pareja porque casi siempre está enfadado/a conmigo. (I feel like I can’t talk to my partner because he or she is almost always angry with me.)
23. Mi pareja me ha forzado a practicar alguna actividad sexual cuando yo no quería. (My partner has forced me to practice some sexual activity when I did not want to.)
24. Mi pareja ha insistido en tocamientos que no me son agradables o que yo no he querido. (My partner has insisted on touching that I am not pleasant or that I have not wanted.)
25. Mi pareja me ha tratado como un objeto sexual. (My partner has treated me like a sexual object.)

Perpetration

1. No he dejado a mi pareja chatear con algunos amigos/as y me he enfadado si lo ha hecho. (I have not let my partner chat with some friends and I have been angry if he/she has.)
2. He hecho a mi pareja eliminar o bloquear amigos/as de sus redes sociales o de su móvil para que no tenga contacto con ellos. (I have made my partner delete or block friends from their social networks or from their mobile phone so that he/she does not have contact with them.)
3. He hecho a mi pareja eliminar comentarios, fotos o vídeos suyos en redes sociales porque me ponían celoso/a. (I have made my partner delete comments, photos or videos of him/her on social networks because they made me jealous.)
4. He controlado o tratado de impedir con comentarios que mi pareja hiciera algo que quería hacer. (I have controlled or tried to prevent my partner from doing something he/she wanted to do with comments.)
5. He intentado que mi pareja no hable o vea s sus amigos y/o familiares. (I have tried to stop my partner from talking or seeing his/her friends and/or family.)
6. He comprobado lo que mi pareja ha hecho y le he exigido que me diga dónde ha estado. (I have checked what my partner has done and have required him/her to tell me where he/she has been.)
7. He preguntado a mi pareja dónde está cada minuto del día. (I have asked my partner where he/she is every minute of the day.)
8. He espiado las cosas de mi pareja (teléfono, correos, redes sociales…). (I have spied on my partner’s things (telephone, emails, social networks ...).
9. He comprobado por amistades, familiares y otras vías si es cierto que mi pareja estaba donde me dijo que estaba. (I have checked with friends, family and other ways if it is true that my partner was where he/she said he/she was.)
10. He vigilado todo lo que mi pareja ha hecho. (I have watched everything my partner has done.)
11. He intentado obtener acceso a la cuenta de red social de mi pareja. (I have tried to access my partner’s social network account.)
12. He acusado a mi pareja de coquetear con otro/a. (I have accused my partner of flirting with another.)
13. Me he enfadado si veo que mi pareja está en línea y no me contesta enseguida. (I have gotten angry if I see that my partner is online and does not answer me right away.)
14. He estado pendiente de si mi pareja estaba en línea en el móvil o conectado/a en las redes sociales. (I have been aware of whether my partner was online on the mobile or connected on social networks.)
15. Me he puesto celoso/a después de leer los mensajes que ha recibido mi pareja en su cuenta o en comentarios en sus fotos. (I have become jealous after reading the messages my partner has received on his/her account or in comments on his photos.)
16. He insultado con frases ofensivas a mi pareja. (I have insulted my partner with offensive phrases.)
17. He insultado o menospreciado delante de los demás a mi pareja. (I have insulted or belittled my partner in front of others.)
18. He criticado, insultado o gritado a mi pareja. (I have criticized, insulted or yelled at my partner.)
19. Mi pareja siente que no puede hablar conmigo porque casi siempre estoy enfadado/a con él/ella. (My partner feels that he/she cannot talk to me because I am almost always angry with him/her.)
20. He forzado a mi pareja a practicar alguna actividad sexual cuando él/ella no quería. (I have forced my partner to practice some sexual activity when he/she did not want to.)
21. He insistido en tocamientos que no le son agradables o que mi pareja no quería. (I have insisted on touching that is not pleasant or that my partner did not want.)
22. He tratado a mi pareja como un objeto sexual. (I have treated my partner as a sexual object.)
